# The effect of familiarity on neural tracking of music stimuli is modulated by mind wandering

**DOI:** 10.3934/Neuroscience.2023025

**Published:** 2023-11-10

**Authors:** Joan Belo, Maureen Clerc, Daniele Schön

**Affiliations:** 1 Athena Project Team, INRIA, Université Côte d'Azur, Nice, France; 2 Aix Marseille University, Inserm, INS, Institut de Neurosciences des Systèmes, Marseille, France; 3 Institute for Language, Communication, and the Brain, Aix-en-Provence, France

**Keywords:** auditory attention detection, stimulus reconstruction, EEG, familiarity, mind wandering, auditory attention

## Abstract

One way to investigate the cortical tracking of continuous auditory stimuli is to use the stimulus reconstruction approach. However, the cognitive and behavioral factors impacting this cortical representation remain largely overlooked. Two possible candidates are familiarity with the stimulus and the ability to resist internal distractions. To explore the possible impacts of these two factors on the cortical representation of natural music stimuli, forty-one participants listened to monodic natural music stimuli while we recorded their neural activity. Using the stimulus reconstruction approach and linear mixed models, we found that familiarity positively impacted the reconstruction accuracy of music stimuli and that this effect of familiarity was modulated by mind wandering.

## 1. Introduction

It is well established now that sounds are represented in the brain at multiple levels, including subcortical [1] and cortical areas [2]. Notably, it has been shown that the amplitude envelope (AE) of a dynamic acoustic stimulus, such as speech or music, is represented at a cortical level via a phase-coupling of neural rhythms onto the temporal structure of the auditory stimuli [3],[4]. More precisely, this coupling takes place in the theta-delta range and also affects the amplitude of high frequency activity in the human auditory cortex [2],[5]–[7]. One way to investigate this cortical tracking of AE of continuous auditory stimuli is to use the stimulus reconstruction approach (SR) which allows reconstructing the AE of an auditory source using neural activity recorded with magneto/electroencephalography [3]. Moreover, SR is interesting to gain insights about the factors that may influence the cortical representation of continuous auditory stimuli, such as speech or music. For instance, studies have investigated the effect of speech intelligibility on the cortical tracking of speech stimuli (e.g., [8]), the impact of musical expertise on speech and music representation (e.g., [9]–[11]) or even the effect of attention in more complex auditory environments on the cortical representation of attended and unattended speech streams (e.g., [12],[13]). However, it is possible that other factors, cognitive as well as behavioral, may impact the cortical representation of sounds [11].

One possible candidate is the familiarity with the stimulus. This idea is supported by studies showing that familiar words [14] and voices [15] are recognized more promptly than unfamiliar ones. This is further supported by other works showing distinct processing for familiar and unfamiliar voices (e.g., [16]). Very recent studies directly investigated the effect of familiarity on the cortical representation of speech and found better cortical representation when listeners are more familiar with the stimuli [8]. While these results are rather consistent for speech stimuli, this is not the case when investigating the role of familiarity on the cortical representation of musical stimuli. Indeed, some studies show, as described with speech stimuli, an enhancement of cortical tracking for familiar compared to unfamiliar song utterances [17] and music [18],[19]. However, other studies show the opposite pattern, meaning a better cortical tracking of unfamiliar music compared to familiar ones [20].

Beyond stimulus familiarity, another potential factor impacting cortical representations of auditory sources is the resistance to internal distractors. One way to address one's ability to resist internal distractors is to estimate the level of mind wandering during a task. While there is no clear definition of mind wandering, one can consider it as periods of inattention (toward the task at hand) during which self-generated thoughts arise (see [21]). Interestingly, it has been shown that when the mind wanders, attention is reduced toward external stimuli, which negatively impacts the processing of external information and the achievement of the external task [22],[23]. Considering that cortical tracking is enhanced when attending to the stimuli (e.g., [12]), periods of inattention toward the auditory stimuli (i.e., mind wandering) may negatively impact its cortical representation.

In light of these considerations, the objective of this study was to investigate to what extent familiarity and mind wandering may predict the reconstruction accuracy of musical ecological stimuli.

## 2. Materials and methods

### 2.1. Participants

A total of forty-one subjects took part in the experiment (28.93 ± 10.94 years, min = 18 years, max = 54 years, 22 females). All the participants included in the analysis reported no history of hearing disorders, attention impairment or neurological disorder and were not under medication.

The experiment was approved by the Operational Committee for the Evaluation of Legal and Ethical Risks (COERLE) of INRIA and was undertaken in accordance with the Declaration of Helsinki. Each participant provided written informed consent and was financially compensated.

### 2.2. Main experimental task

#### 2.2.1. Stimuli

Monodic stimuli consisted of 60-second recordings of four well-known and four less-known classical piano pieces. The well-known musical excerpts were taken from Beethoven's “Für Elise,” Bach's “Prelude in C Major,” Mozart's “Sonata in C Major,” K545, and Beethoven's “Pathétique” Sonata. The less-known musical excerpts were taken from Beethoven's “Lustig und Traurig,” Scarlatti's “Sonata in A Minor,” K54, Beethoven's “Sonata in F Presto” and Bach's “Prelude No. 8.” All musical excerpts were RMS normalized. Stimuli were presented via Sennheiser HD-25 supra-aural headphones at a comfortable level.

#### 2.2.2. Procedure

Participants were instructed to concentrate their attention on the musical excerpt as if they were trying to memorize it. While the musical excerpt was played for 60 seconds, a fixation cross was displayed at the screen center. Each of the musical excerpts was presented twice for a total of sixteen 60-second trials. The pseudo-random order of presentation avoided direct repetition of a given excerpt. The whole experiment was monitored using a custom version of OpenSesame developed by Oticon Medical [24].

#### 2.2.3. Behavioral indicators

At the end of each trial, the participants were asked several questions (see table 1).

**Table 1.** Questions asked to the participants after each excerpt and the corresponding behavioral indicator.

**Questions**	**Possible answers**	**Behavioral indicator**
*Do you know this music?*	*Yes / No / Not sure*	Familiarity
*How much did your mind wander?*	*0-25% / 25-50% / 50-75% / 75-100%* (of the time)	Mind Wandering

For *Familiarity* we computed two categories across stimuli: *Low* and *High familiarity*. The *Low familiarity* category combines the answers *No* and *Not sure* while the *High familiarity* category is composed of the answer *Yes*. Importantly, because the musical excerpts were presented twice during the experiment, we only used the answer to the first presentation of each stimulus to avoid a repetition bias.

For *Mind Wandering* we computed two categories across participants: *Low* and *High Mind Wandering*, corresponding to participants for which the average mind wandering was lower or higher than the grand mean, respectively.

In addition to these behavioral indicators created from the questions, because each musical excerpt was presented twice, we also extracted a *Repetition* indicator, composed of two categories (i.e., *First Presentation* and *Second Presentation* of the stimulus). Importantly, this indicator allowed us to explore the effect of the acquired (short-term) knowledge on neural tracking.

#### 2.2.4. EEG data

Electroencephalography data were recorded using an ANT Refa8 amplifier and a 21-electrode cap (arranged in accordance with the International 10–20 system) with a sampling rate of 256 Hz and an average reference. They were preprocessed using the *MNE-python* package [25] according to the procedure described in [26]. First, raw EEG data were cut into 60 s epochs, each epoch representing a trial, and the first 500 ms of each epoch was discarded to avoid modeling the response to the stimulus onset. Next, EEG data were digitally filtered between 1–40 Hz, and bad channels were interpolated via spherical spline interpolation when necessary. Then, ICA was performed to remove eye-blinks and saccade artifacts. Afterward, EEG data were digitally filtered between 1–9 Hz [27],[28] using a 4th order Butterworth zero-phase-shift filter and downsampled to 64 Hz. Finally, 60 s epochs were cut into 30 s epochs, resulting in 32 epochs with the aim of having enough data to train and test the stimulus-reconstruction model.

#### 2.2.5. Audio feature extraction

Amplitude envelopes of the audio monodic stimuli were obtained using the function *human_cochleagram* from the Python *Pycochleogram* package [29]. This function allows 1) computing an equivalent rectangular bandwidth (ERB) filter bank and 2) using this filter bank to decompose the signal into subband envelopes. Afterwards, subband envelopes were averaged to obtain a unique envelope. Each envelope was then digitally filtered between 1–9 Hz with a 4th order Butterworth zero-phase-shift filter, downsampled to 64 Hz and cut into 30 s long epochs in order to match EEG preprocessing.

#### 2.2.6. Stimulus reconstruction

**Figure 1.**
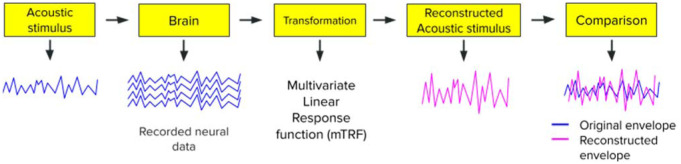
Illustration of the stimulus reconstruction approach. First, participants listen to an acoustic stimulus (i.e., a musical excerpt) while their neural activity is recorded using an EEG. Then, a transformation (i.e., a multivariate temporal response function) is applied to the recorded neural activity in order to reconstruct the original acoustic stimulus. Afterward, a comparison (i.e., a correlation) is computed between the original acoustic envelope and the reconstructed one to estimate the reconstruction quality (i.e., the reconstruction accuracy).

In this study, we used the same stimulus reconstruction approach as [30] (this approach is also described in several other articles; see [26] and [31] for a comprehensive description and see Figure 1 for an illustration).

### 2.3. General procedure

The stimulus reconstruction approach allows one to reconstruct an estimate of the envelope of the auditory stimulus *s* using electrophysiological neural data *d* via a linear reconstruction model *g* [30]. The reconstruction model *g(τ, n)* is a temporal response function (see [32] for details) that maps neural data *d(t, n)* to stimulus *s(t)* as follows:

$$\hat{\text{s}}\left(\text{t}\right)=\underset{n}{\sum^{\text{​}}}\overset{\tau }{\underset{}{\sum^{\text{​}}}}d\left(t+\tau ,n\right)g\left(\tau ,n\right)$$

where *ŝ* denotes the reconstructed stimulus, *d(t,n)* represents the response of electrode n at time t=1 ... T, and *τ* are some time lags that represent a window in which the brain's response to the stimulus is supposedly optimal. We defined *τ* to go from 0.200 ms pre-stimulus to 0.350 ms post-stimulus based on previous works [30],[38]. At a sampling rate of 64 Hz, this corresponds to 36 sample shifts (incl. the zero shift).

The model g is estimated by minimizing the mean-squared error between the original stimulus *s(t)* and the reconstructed one *ŝ(t)*:

$$\min e=\underset{t}{\sum }{\left[s\left(t\right)-\hat{\text{s}}(\text{t})\right]}^{2}$$

A robust minimizer of the mean-squared error is obtained using the following matrix operations:

$$g={\left(D^{T}D+\lambda I\right)}^{-1}D^{T}s$$

where *D* is the lagged time series of the response matrix *d*, and *λ* is a ridge parameter term introduced to avoid overfitting. Importantly we used a multivariate approach, fully exploiting the 21 EEG channels to reconstruct the stimulus. The model parameters as well as the ridge parameter are generally estimated using the leave-one-out cross validation (LOO) procedure. Once the model parameters have been tuned, it can be tested on new data (generally, the left out fold), and the end of this process is the reconstructed stimulus *ŝ*. Finally, a reconstruction score is computed, to estimate the reconstruction accuracy of the model, as the Pearson correlation between the reconstructed stimulus ŝ and the original stimulus *s*. The higher the reconstruction score is, the better the reconstruction accuracy.

### 2.4. Training and testing the model

In this study, we wanted to see if we can reconstruct a trial with a model trained on every other monodic trial. To do so, we used a custom Python script where an initial LOO was performed to separate training and test phase; and because we introduced a ridge parameter (λ), we used a second LOO nested in the training phase to estimate the optimal value of the ridge parameter.

At the end of the test phase, a reconstruction ŝ_i_ is obtained for each musical excerpt s_i_, based on a model trained on all the other musical excerpts. This procedure allowed us to make sure that the musical excerpt we wanted to reconstruct was only used to validate the model.

Because we had 32 monodic trials, we trained separate models for each trial type. Therefore, this resulted in 32 models for monodic trials for each participant.

### 2.5. Reconstruction accuracy

The reconstruction accuracy corresponds to the Pearson correlation between the reconstructed envelopes and the original musical envelopes. This led to 32 r values per participant.

### 2.6. Statistical analysis

In order to see whether the behavioral indicators were good predictors of the reconstruction accuracy of monodic stimuli, we fitted linear mixed models (LMMs) on single trial data using R statistical software and the lme4 package [33]–[36]. To take into account the measures dependency induced by our experimental design, LMMs included Subject and Stimulus as random effects. The model assumptions of non-collinearity of the predictors as well as the linearity, homoscedasticity and normality of the residuals were systematically verified using visual inspections of the residuals QQ-plot and histograms. In addition, we reported 95% confidence intervals (CIs), and p-values were computed using a Wald t-distribution approximation.

## 3. Results

Participant responses concerning the familiarity of the musical excerpts were rather consistent with our initial stimulus categorizations of familiar versus unfamiliar music. Participants answered unfamiliar at 67% when the stimuli were unfamiliar, and they answered familiar at 80% when the stimuli were familiar. Because there was no systematic categorization of musical excerpts across participants, we used individual participants' responses to categorize musical excerpts as familiar or not.

The overall reconstruction accuracy, namely, the correlation between the EEG-reconstructed envelopes and the original musical envelopes, was 0.063 (± 0.08). This estimate was significantly different from the null distribution (*t(40) = 14.32, p < 0.001; Cohen's d = 2.24*).

In order to explore the predictive power of the behavioral indicators (familiarity, mind wandering and repetition) on the reconstruction accuracy of monodic trials, we started by computing a stepwise backward regression (SBR). This model selection procedure involves starting with a model containing all the possible predictors (the “full model”) and then iteratively removing the least useful predictor one-at-a-time until no further predictor can be deleted without a statistically significant loss of fit. Therefore, our full model was a model with Stimulus and Subject as random effects and Familiarity, Mind wandering and Repetition, as well as all the possible interactions between them, as fixed effects (Formula: Reconstruction ~ Familiarity * Mind wandering * Repetition * (1|Subject) + (1|Stimulus)).

The SBR indicated that the best model to predict the reconstruction accuracy of monodic trials was a model with Stimulus and Subject as random effects and Familiarity as fixed effect (Formula: Reconstruction ~ Familiarity + (1|Subject) + (1|Stimulus); see Table 2 for full details).

**Table 2.** Summary table for the stepwise backward regression model.

**Backward reduced random-effect table**							
	Eliminated	npar	logLik	AIC	LRT	Df	Pr (>Chisq)
<none>		7	1462.6	-2911.2			
Subject	0	6	1428.6	-2845.3	67.946	1	< 2.2e-16 ***
Stimulus	0	6	1457.7	-2902.1	11.098	1	0.0008642 ***

**Backward reduced fixed-effect table** *degree of freedom method: Satterthwaite*							
	Eliminated	Sum Sq	Mean Sq	NumDF	DenDF	F-value	Pr (>F)

Familiarity*Mind wandering*Repetition	1	0.0037	0.0037	1	1294.83	0.634	0.425
Mind wandering*Repetition	2	0.00062	0.00062	1	1283.22	0.106	0.744
Familiarity*Repetition	3	0.0021	0.0021	1	1297.88	0.36	0.548
Repetition	4	0.00683	0.00683	1	1228.62	1.173	0.278
Familiarity*Mind wandering	5	0.01955	0.01955	1	1280.28	3.357	0.067 .
Mind wandering	6	0.00061	0.00061	1	39	0.105	0.747
Familiarity	0	0.03773	0.03773	1	454.34	6.4675	0.011 *

**Figure 2.**
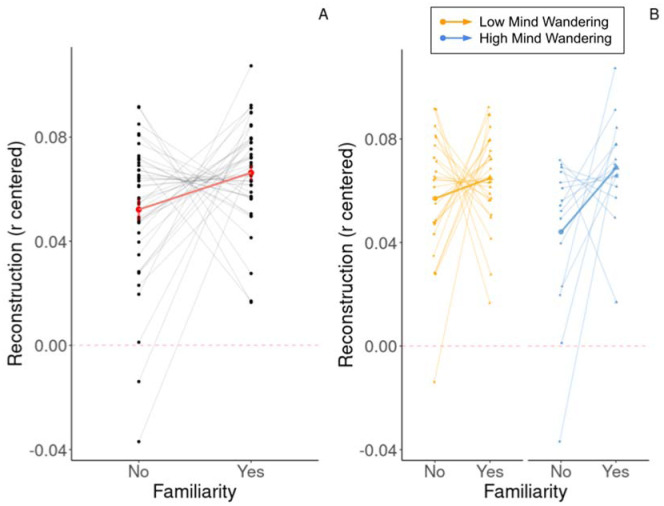
Illustration of the effect of familiarity on reconstruction accuracy and the effects of Familiarity on reconstruction accuracy within the Mind Wandering indicators' levels. A) Average reconstruction accuracy when excerpts were familiar and unfamiliar. Average reconstruction was higher for familiar than unfamiliar excerpts (*β* = 0.01, 95% CI [3.02e−03, 0.02], p = 0.011). B) Average reconstruction when excerpts were familiar and unfamiliar and when mind wandering was low or high. Average reconstruction was higher for familiar excerpts for participants with high mind wandering (*β* = 0.02, 95% CI [0.04, 8.64e−03], p = 0.003), but it was similar for familiar and unfamiliar excerpts for participants with low mind wandering (*β* = −6.96e−03, 95% CI [−0.02, 5.19e−03], p = 0.262). Error bars indicate plus or minus one standard error of the mean. Large points indicate the group averages, and small points indicate individual data.

The best model clearly differed from a “null model” containing only the random effects (Likelihood Ratio Test: Chisq = 6.5, p = 0.01). Importantly, the best model indicated that familiarity affected the reconstruction accuracy of monodic stimuli (*β = −0.01, 95% CI [3.02e−03, 0.02], t(1307) = 2.54, p = 0.011; Std. beta = 0.16, 95% CI [0.04, 0.29]*). More precisely, reconstruction of familiar stimuli was higher than reconstruction of less familiar ones (see Figure 2A). This model significantly predicted around 11% of the variance of the reconstruction score (*conditional R² = 0.11*). However, the part of explained variance related to the fixed effect (Familiarity) alone was around 0.7% (*marginal R²=6.52^−3^*) which means that the vast majority of the variance explained by this model is due to the random effects alone, namely, the Stimulus and the Subject.

Moreover, the SBR indicated a trend towards an interaction between the Familiarity and the Mind wandering (see Figure 2B). Therefore, we computed a LMM for each level of the Mind wandering Predictor to better understand the behavior of the effect of stimulus familiarity.

We found a significant effect of familiarity on the reconstruction of monodic stimuli for the High Mind wandering level (*β* = −0.02, 95% CI [−0.04, −4.95e−03], t(475) = −2.54, p = 0.011; Std. beta = −0.26, 95% CI [−0.47, −0.06]) but not for the Low Mind wandering level (*β* = −0.01, 95% CI [−0.02, 2.57e−03], t(827) = −1.57, p = 0.118; Std. beta = −0.13, 95% CI [−0.29, 0.03]). Indeed, the reconstruction accuracy of monodic stimuli for the participants who experienced more mind wandering was higher for familiar stimuli than for less familiar ones (see Figure 2B), while this was not the case for the participants who experienced less mind wandering (see Figure 2B)

## 4. Discussion and conclusion

In this work, we hypothesized that reconstruction accuracy would be higher for familiar excerpts compared to less familiar ones. Our analysis confirmed this hypothesis and revealed a positive main effect of familiarity on reconstruction accuracy (Figure 1A). This result corroborates the findings of previous works showing an enhancement of cortical tracking for familiar song utterances [17] and familiar natural music [18],[19]. One possible reason why the cortical tracking of familiar stimuli is enhanced compared to unfamiliar ones is because they are more predictable, as they elicit more precise expectations due to the presence of preexisting memory traces [24],[37]. Some recent results suggest that expectations may directly impact cortical tracking of music and that, in particular, long-term experience better captures the neural correlates of melody compared to short-term statistical regularities [38],[39]. It has also been shown that stimulus predictability could have an influence on auditory scene analysis (e.g., [40]), although most of the studies investigating the influence of predictability on auditory scene analysis used paradigms in which concurrent sounds were present or where sound sequences contained deviant sounds.

Interestingly, one may argue that unfamiliar stimuli could have elicited greater neural responses, leading to better cortical tracking, because they are more prone to induced expectations violation than familiar ones, which could be in line with the results of Kumagai and colleagues [20]. However, extending the proposition of Weineck and colleagues [19], it is likely that our unfamiliar stimuli did not elicit expectation violation because they were classical Western music excerpts that were composed following the common rules of the genre. Thus, even if participants were not familiar with the particular excerpt, they were probably familiar with Western music in some way. Consequently, it is probable that familiar music excerpts, similarly to more intelligible or familiar speech (e.g., [41],[42]), induced better cortical tracking because memory priors were higher compared to unfamiliar music and because unfamiliar music excerpts did not induce violation of these priors.

Besides an explanation in terms of better predictions for familiar stimuli, it is also possible that familiarity modulated cortical tracking because familiar music excerpts elicited greater engagement/attention of the participants.

That being said, while the effect of familiarity was significant, its predictive power was rather weak (0.7% of the reconstruction accuracy), suggesting that, in our experiment, the familiarity with the musical excerpt probably does not massively impact its cortical tracking. However, because most previous papers in the field do not report the predictive power, it remains difficult to assess the global impact of familiarity on cortical tracking.

Here, we also hypothesized that the mind wandering level will negatively affect the reconstruction accuracy of monodic excerpts. Inconsistent with our hypothesis, we found that reconstruction accuracy was similar for all the participants, no matter if they experienced a low or a high level of mind wandering during the experiment. Although this result may sound surprising because of the detrimental effect of mind wandering on task performance (e.g., [43]), we used a retrospective measurement of mind wandering requiring the participants to estimate how frequently their mind wandered during each trial. This approach may suffer from forgetting and mental-aggregation biases [44].

The original result of this work is that the effect of familiarity was modulated by the global level of mind wandering. Indeed, participants that experienced a high level of mind wandering during the experiment yielded higher reconstruction accuracy for familiar excerpts than for unfamiliar ones. By contrast, participants that experienced a low level of mind wandering did not show such a familiarity effect. This could indicate that when attention is mostly dedicated to the task at hand (i.e., listening to the musical excerpt), familiarity may not enhance the cortical tracking, possibly because a large amount of the available attentional resources can be devoted to actively listening to the excerpts, independently of whether it is familiar or unfamiliar. However, when attention is directed to unrelated thoughts rather than to the task at hand, leading the target musical excerpt to become “unattended,” the familiarity may compensate for this reduction of attentional resources devoted to the treatment of the musical excerpt. In such a context, familiar excerpts seem to somehow better “capture” attentional resources, leading to a more robust cortical representation. This idea could find support in some results suggesting that 1) although the underlying mechanism is still unclear, a cortical representation of the unattended sound can occur without direct attention (see, for example, [12] and [45]), and 2) familiar excerpts, because they are more predictable, are less computationally demanding [37], which suggests that they may be processed with less attentional resources.

Interestingly, we found that the reconstruction accuracy was similar for the first and the second presentation of the musical excerpts, indicating a lack of short-term knowledge effect on cortical representation of monodic musical stimuli. This result is in line with the work of Di Liberto and colleagues [9], who showed no effect of repetition on cortical tracking of monodic synthetic music, but not with the work of Madsen and collaborators [18], who found a decrease in cortical tracking for familiar music across repetitions.

Overall, our results indicate that stimulus familiarity favors neural tracking. Moreover, this familiarity effect is present with long-term knowledge but not with short-term knowledge of the stimuli. Importantly, this long-term familiarity effect on neural stimuli representations is modulated by attention and is maximal when participants are not well focused on the task at hand.

Further work is required to better understand how this effect of familiarity on cortical representation is driven by long-term knowledge about the musical excerpts.
